# Supplementary material for: Rapid turnover of life-cycle-related genes in the brown algae
Source: Genome Biol. 2019 Feb 14;20:35. doi: 10.1186/s13059-019-1630-6 (PMC6374913; doi:10.1186/s13059-019-1630-6)
Supplement: Supplementary file 3 — Protein alignments, corresponding Gblocks html file and CDS sequences for the lineage conserved gametophyte-biased genes. (ZIP 2109 kb) [file 13059_2019_1630_MOESM3_ESM.zip › Con_GA_alignments/SJ04982.aa_ali.fasta-gb.htm]

SJ04982.aa.fasta.trans.aa\_ali.fasta


## Gblocks 0.91b Results

Processed file: **SJ04982.aa.fasta.trans.aa\_ali.fasta**  
Number of sequences: **2**  
Alignment assumed to be: **Protein**  
New number of positions: **135** (selected positions are underlined in blue)

```
                         10        20        30        40        50        60
                 =========+=========+=========+=========+=========+=========+
SJ04982          MVVLHVRIFTHRRGVDKDHAFLINSMPGGVSERLDELKVDENTTFRRLRRMIELNEDRGM
Mac.58719.1      ------------RGVNKDHAFLINSMPGGVSERLDELKVDENTTFRRLRRMIELNEDRGM
                             ################################################


                         70        80        90       100       110       120
                 =========+=========+=========+=========+=========+=========+
SJ04982          MRRTPLFQELLFIMINSSNPHNYGKDLRYRYRLGVMNEDRGSDIPRLVSMEHEQSDMPVL
Mac.58719.1      MRRTPLFQELLFVMINSSNPHNYGKDLRYRYRLGMLIEDEGSQIPKLVPMEHEQSYMPVL
                 ############################################################


                        130       140       150       160       170       180
                 =========+=========+=========+=========+=========+=========+
SJ04982          DYYRNTARLDLVLTPSHRALRQHPGNNRLLLSMLRSTMLEGNDGMPRQGLWSTIGYSAAA
Mac.58719.1      DYYRNTARLDLVLVPAT-------------------------------------------
                 ###############                                             


                        190       200       210       220       230
                 =========+=========+=========+=========+=========+=====
SJ04982          ARATVAPAGVATVLAMASHGKPCGHMSTARCHGMARGGNDRDRTHGKSHGKPPT-
Mac.58719.1      ---QISPSGVCT----------------------SNPDSEPPRSQSASGKQVATD
                                                           ############
```

```
Parameters used
Minimum Number Of Sequences For A Conserved Position: 2
Minimum Number Of Sequences For A Flanking Position: 2
Maximum Number Of Contiguous Nonconserved Positions: 8
Minimum Length Of A Block: 10
Allowed Gap Positions: None
Use Similarity Matrices: Yes
```

```
Flank positions of the 2 selected block(s)
Flanks: [13  135]  [223  234]  

New number of positions in SJ04982.aa.fasta.trans.aa_ali.fasta-gb:  135  (57% of the original 235 positions)
```
